# A Mouthful of Genomic Data: Single-Cell Insights into Salivary Gland Biology and Disease

**DOI:** 10.3390/biology15080641

**Published:** 2026-04-18

**Authors:** Theresa Wrynn, Satrajit Sinha, Rose-Anne Romano

**Affiliations:** 1 Department of Oral Biology, School of Dental Medicine, State University of New York at Buffalo, Buffalo, NY 14214, USA; 2 Department of Biochemistry, Jacobs School of Medicine and Biomedical Sciences, State University of New York at Buffalo, Buffalo, NY 14203, USA; ssinha2@buffalo.edu

**Keywords:** single-cell RNA-sequencing, salivary glands, regeneration, disease, development, transcriptomics

## Abstract

Single-cell RNA-sequencing (scRNA-seq) studies over the past several years have provided unprecedented resolution into the transcriptomic landscape of both major and minor salivary glands. This technology enables the identification of diverse and functionally specialized cell populations that underlie glandular architecture and physiology. Increasingly, scRNA-seq has become an integral component of experimental design, used not only to validate prior observations but also to uncover novel cell types, pathways, and molecular regulatory mechanisms. As a result, a growing number of publicly available datasets now encompass a wide spectrum of biological contexts including homeostasis, disease, and regeneration. However, inconsistencies in data processing and incomplete reporting of experimental methods pose challenges for reproducibility and limit the ability to distinguish high-quality datasets. As single-cell technologies continue to evolve and become more accessible, their application in salivary gland research is expected to expand, offering deeper insight into both basic biology and clinical translation. This review compiles and summarizes findings from a growing body of scRNA-seq studies of the salivary glands, highlights current limitations, provides methodological considerations, and expounds on key cellular and genomic discoveries to help guide future investigations.

## Simple Summary

Salivary glands are complex exocrine organs that rely on intricate intercellular communication to maintain tissue homeostasis and to respond to tissue damage and disease. Recently, single-cell RNA sequencing has revolutionized our understanding of these organs by providing high-resolution maps of gene expression that define the constituent cell populations. While ever-increasing genomic and epigenomic datasets now characterize salivary gland development, adult tissue maintenance and regeneration, and human pathologies like Sjögren’s Disease, the sheer volume of published data remains a challenge to navigate. This review highlights the power and limitations of single-cell RNA sequencing-based studies and synthesizes key findings from the current single-cell RNA sequencing literature in the salivary gland field. These findings are integrated with available biological knowledge based on genetic, molecular and cellular studies and highlight convergent genomic themes that underpin cell-to-cell communications and the molecular mechanisms driving disease. By identifying critical knowledge gaps and consolidating existing evidence, this review provides a strategic framework to guide future research in the field of salivary gland biology and regenerative medicine.

## 1. Introduction

The salivary gland (SG) is a vital exocrine organ essential for oral health, supporting functions such as lubrication, digestion, and protection against infection [1,2]. Humans and rodents each possess three major pairs of SGs—the submandibular (SMG), parotid (PG), and sublingual (SLG), as well as numerous minor glands. Owing to their physiological similarities to humans, rodents, particularly mice, have long served as valuable models for investigating SG biology and developing potential therapeutic strategies [3]. Structurally, the SG is an arborized epithelial-rich structure comprising secretory acini, contractile and progenitor myoepithelial/basal cells, and branched ductal networks that generate and transport saliva to the oral cavity. Although all three major glands produce and secrete saliva, the composition of their secretions varies. The PG primarily produces a serous, enzyme-rich fluid, while the SLG secretes mucin dense saliva, and the SMG generates a mixed serous–mucous saliva [4]. Damage or dysfunction of the SGs can have profound consequences, leading to an increased risk of periodontal disease, oral pain, speech difficulties, and difficulty in swallowing, all of which significantly impact patients’ quality of life [5,6]. Currently there are no treatments that can fully restore glandular function, and available therapies, such as saliva substitutes, offer only temporary relief, without addressing the underlying root cause. Thus, gaining a deeper understanding of the cellular diversity and molecular mechanisms governing SG function is essential for developing regenerative therapies.

While traditional genetic approaches such as gene knockouts and lineage-tracing studies have been invaluable in elucidating key biological processes, advances in next-generation sequencing have now allowed for more comprehensive genomic profiling of organs and tissues. In this regard, scRNA-seq has been a powerful tool for investigating both homeostatic and diseased states by capturing transcriptomic differences at the level of individual cells and revealing the complex communication between cell types among myriad cells that populate and drive tissue development, maintenance, and homeostasis. Additionally, this approach has offered new insights into the underlying complexity of cellular heterogeneity and identifying rare yet functionally crucial cell types; these findings have unleashed new avenues of follow-up research including those in the field of SG biology.

In recent years, scRNA-seq has become increasingly accessible and cost-effective [7]. Alongside the availability of user-friendly analytical pipelines and advancements in AI (Artificial Intelligence), most laboratories can now readily implement and interpret this sophisticated technology. This growing accessibility has led to a surge in SG-based scRNA-seq studies, beginning with the first report in 2018 [8] and rapidly expanding in the years since with 36 and counting published studies to date (Supplementary Table S1). While these studies have allowed for unprecedented insights and discoveries, it is important to stress that evolving technological and computational improvements will continue to revolutionize and advance our understanding of this complex tissue.

The goal of this review is to highlight major discoveries and the rapidly expanding insights into SG biology and disease made possible through the application of scRNA-seq. Key technical considerations, inherent limitations, and challenges with deriving biologically meaningful interpretations from these datasets are also discussed. Additionally, an extensive literature search was conducted to identify pivotal scRNA-seq-focused studies in the salivary gland field (Supplementary Figure S1). Collectively, the single-cell perspective of the SG transcriptome as highlighted in this review has provided a more refined and comprehensive image of the SG, illuminating the molecular pathways that govern its development, tissue homeostasis, and pathological states.

## 2. Technical Considerations of Single-Cell Data

### 2.1. Tissue Sample Preparation

**Figure 1:**
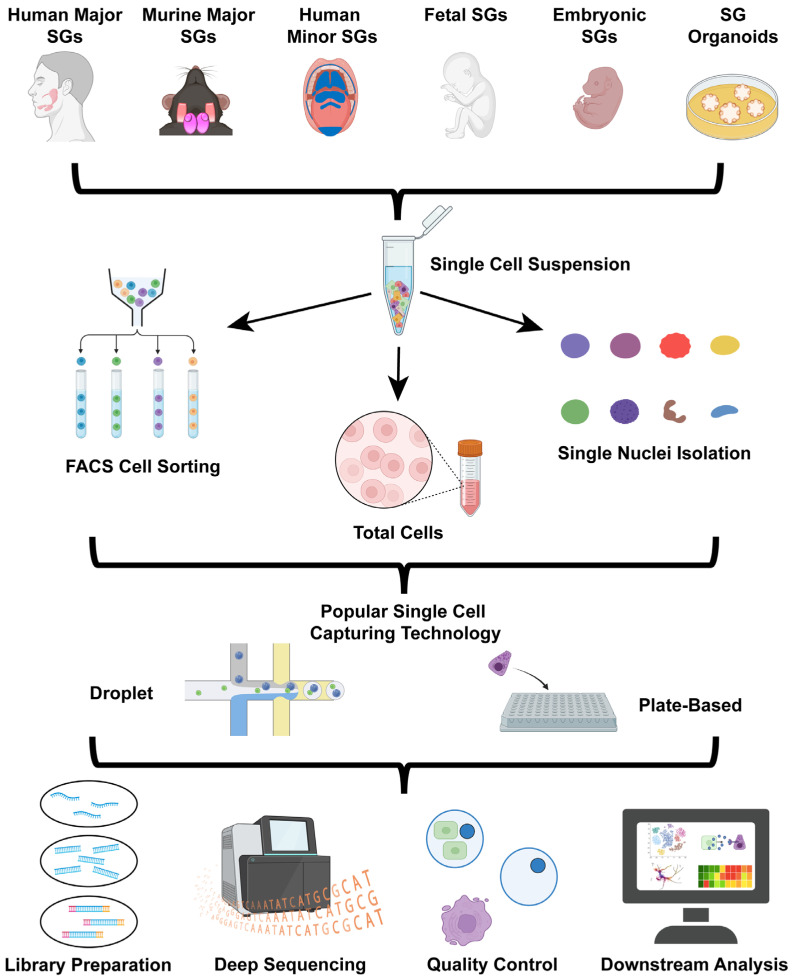
Overview of the workflow to generate salivary gland scRNA-seq data. Illustration showing the diverse range of salivary gland-related starting material which is dissociated into single-cells. Total single cells can be utilized while further sorting using methods such as FACS (fluorescence-activated cell sorting) or single nuclei isolation can be performed. Popular single cell sequencing technology within the salivary gland field is shown along with library preparation, sequencing, and quality control metrics (removing doublets, empty droplets, and apoptotic cells) preparing the data for downstream analysis. Images created in BioRender.

The first and most critical step in the scRNA-seq workflow is sample preparation. In the case of the SGs, scRNA-seq datasets represent a diverse range of tissues, including adult human minor and major salivary glands, murine major salivary glands, human fetal and mouse embryonic SGs, as well as salivary gland-derived organoids (Figure 1). To generate a high-quality scRNA-seq dataset, it is crucial that the tissue/sample is dissociated into a suspension of viable single cells. This is typically achieved through enzymatic [9], mechanical [10,11], or combined dissociation methods [12]. A combination approach can help to overcome the limitations of a single method (enzymatic or mechanical); however, it is essential that the dissociation method chosen is aligned with the goals of the experiment, such as the source of the sample tissue or organoids for, e.g., enrichment for a certain cell population, preserving cell surface markers, or maintaining a specific cellular function [13,14]. Recent advances, such as the hypersonic levitation and spinning (HLS) method, which utilizes high-frequency acoustic soundwaves to dissociate the tissue while maintaining cell integrity and viability [15], can be valuable for generating more physiologically relevant single-cell data.

After tissue dissociation, subsequent enrichment can be performed to capture specific cell populations of interest. For instance, fluorescence-activated cell sorting (FACS) is a widely employed technique to separate fluorescently labeled subpopulations, while other enrichment strategies include centrifugation, bead-based isolation, and microfluidic sorting. Maintaining a minimum cell viability of 70%, but ideally 90% [16], and integrity throughout this process is essential; however, it is important to keep in mind that tissue dissociation often leads to cell loss and cellular stress, leading to transcriptional alterations that represent a major source of variability [17]. Prior to proceeding with sequencing, it is critical to access cell viability and ensure minimal aggregation, as cell clumping can compromise downstream analyses.

### 2.2. Isolating Single Cells and Library Preparation

Once the sample has been properly dissociated, various high-throughput technologies can be employed to capture individual cells, enabling the analysis of hundreds to tens of thousands of cells simultaneously. Commonly used commercially available platforms include droplet-based 10X Chromium and the microwell-based BD Rhapsody system [18,19]. The scRNA-seq workflow of both these platforms allows for robust single-cell isolation and capture. Subsequent steps after cell isolation and capture include sample barcoding, reverse transcription of the mRNAs, cDNA amplification and follow-up preparation. One key aspect of these molecular steps is the incorporation of unique molecular identifiers (UMIs) which enable accurate quantification of individual transcripts. Sequencing of the cDNA library is subsequently performed using Next Generation Sequencing (NGS) platforms such as Illumina, Pacific Biosciences, and Oxford Nanopore [20]. These third-generation sequencing technologies enable high-throughput and long-read sequencing capabilities generating billions of reads per run. Pacific Biosciences employs single-molecule, real-time (SMRT) sequencing using fluorescently labeled nucleotides to read DNA fragments, whereas Oxford Nanopore determines nucleotide sequences by measuring changes in ionic current as DNA strands pass through nanopores [20]. Illumina sequencing, in contrast, is based on a sequencing-by-synthesis approach that utilizes reversible dye-terminators to identify incorporated nucleotides with high accuracy and has the capabilities to sequence the entire DNA segment. After sequencing, raw data must be processed and the quality assessed prior to downstream analyses to ensure reliability and reproducibility of results.

### 2.3. General Guidelines for Quality Control

scRNA-seq datasets are generated using a wide range of experimental platforms and analytical pipelines, each with distinct standards that can lead to considerable variation in data quality. There are several factors that can influence the quality of these datasets, including tissue dissociation protocols, number of cells captured, sequencing depth, and the sequencing platform used. For instance, poor cell viability during tissue dissociation or lysis of single cells can compromise data integrity. Additionally, challenges such as low mRNA recovery, inefficient cDNA synthesis, and high mitochondrial transcript contamination can further diminish data quality. Prior to downstream analyses, it is essential to filter out low-quality cells using computational tools designed for this purpose. While comprehensive reviews centered on quality control (QC) [21,22,23,24,25,26] strategies are available, it is imperative to highlight key concepts that are particularly important to consider when interpreting scRNA-seq results.

A critical QC parameter to evaluate is the proportion of mitochondrial gene transcripts as this serves as a proxy for cell viability and an indicator of dying or low-quality cells [23]. Although standardized bioinformatics pipelines such as Seurat [27] provide default thresholds, many laboratories opt to use different and arbitrary cutoffs: commonly around 5% or 10%. However, default values may not be suitable across different species, tissues, or even cell types being assessed [25]. This represents a delicate balance as an overly permissive threshold can result in low-quality or apoptotic cells remaining in the analysis, whereas stringent cutoffs risks eliminating unrecovered cellular compositions and the loss of rare but biologically meaningful cell populations leading to misinterpretation of the data. Therefore, it is essential that studies include metrics such as mitochondrial filtering thresholds when publishing scRNA-seq data to ensure transparency and reproducibility.

In addition, technical and biological variability should also be assessed. Among these, cell-cycle-associated variation is frequently underreported despite its clear effects on gene expression profiles and the fact that it can mask subpopulations when not removed [28]. While most datasets evaluate mitochondrial contamination as part of the Seurat workflow, cell cycle effects are often overlooked. As cells prepare to progress through the cell cycle, their total RNA content fluctuates and can be different compared to their non-cycling counterparts, which can obscure true biological differences between cell populations if unaccounted for [29]. Adjusting for cell cycle effects can reveal otherwise hidden insights such as cell differentiation trajectories or a discrete subpopulation that might otherwise be misinterpreted and may be important depending on the aim of the study [29]. Even in slowly proliferating tissues such as the SG [30,31], cell cycle effects could be one source of variation. Notably, proliferative differences have been identified between adult male and female murine SMG [32]; this needs to be taken into account when comparing between the sexes in rodents. Cell cycle scoring tools from Seurat [32] or other tools such as ccRemover [33] can help investigators examine any potential impact of the cell cycle on scRNA-seq analysis and to eliminate bias, if any, from the analysis.

Batch effects represent another major source of confounding variation. These arise from technical inconsistencies, such as differences in sample processing time, reagent lots, or sequencing platforms, rather than inherent biological factors [29]. If left uncorrected, batch effects can lead to misleading interpretations [34]. Integration frameworks such as Harmony, LIGER, and Seurat offer robust methods to mitigate these issues to recover biologically meaningful results [35]. In summary, rigorous and transparent reporting of QC metrics, including mitochondrial content, cell cycle considerations, and batch correction, are essential for reproducibility and for accurately reflecting the biological state of the tissue, such as in salivary gland tissues.

### 2.4. Annotating scRNA-Seq Datasets

Cell type characterization has evolved from an initial labor-intensive, complex process with limited molecular markers to a more streamlined and automated approach. This is primarily due to a broader understanding of cell type-specific markers, increased cell capture efficiency, and improved sequencing depth. Annotation can be performed with manual methods, computationally, or through an integrated approach to achieve accurate cell classifications. This step is pivotal, as it enables the identification of the full range of cell populations, including rare cell types and provides the foundation upon which subsequent biological interpretations are built. One of the most widely used strategies for defining cell populations is the identification of “marker genes” which represent genes enriched in each specific cluster. Accurate annotation using this method depends heavily on the current knowledge of established markers across cell types. It is thus imperative that publications provide details of gene expression plots so that readers can assess how accurately the cell cluster label fits with the spatial distribution of the respective cell marker. Ideally, multiple markers are employed to reliably delineate a cell population [36] especially when evaluating rare cell populations. Publicly available databases such as PanglaoDB [37], CellMarker [38], SCsig, CellMatch [39], and celldex [40] can be leveraged to identify additional markers to support classification while algorithms such as NS-Forest [41], scGeneFit [42], and COMET [43] can aid in predictive marker gene selection. Nevertheless, challenges remain. Some markers are shared among multiple cell types, which can complicate annotation. For instance, Nkcc1 and Aqp5 are enriched in all acinar cells subtypes of SGs, but also show expression in the intercalated ductal cell population [8,44,45,46,47,48]. This overlap underscores the importance of using multiple markers to accurately assign cell identity. Another notable example of this complicating issue is distinguishing myoepithelial (MECs) from basal cells as both cell types exhibit similar transcriptional profiles and hence may cluster together. In such cases, deeper sub-clustering and a closer examination are often necessary, as both cell types express *Trp63*, *Krt14*, and *Krt5* [49,50,51,52,53,54,55] while only MECs express *Cnn1*, *Acta2*, and *Myh11* [51,56,57]. In the case of such a nuanced situation, manual annotation offers an advantage, as it allows researchers to incorporate expert judgment and contextual interpretation, whereas automated computational methods might fail to detect subtle differences in the gene expression profiles of cell populations. However, manual annotation approaches can be subjective and must be thoroughly documented to ensure reproducibility and transparency.

While the repertoire of validated markers in humans remains more limited compared to mice, resources such as the Human Protein Atlas (www.proteinatlas.org) can be valuable for identifying additional human markers. However, it is important to note that mRNA and protein expression levels do not always correspond directly, despite significant overall correlation between the two [58,59,60,61,62]. Similarly, the Human Cell Atlas (www.humancellatlas.org), a collaborative consortium of atlases that includes SG scRNA-seq datasets [63], can be leveraged to support supervised learning approaches for annotation. Supervised learning enables the projection of cell type labels from a well-characterized reference dataset to an unlabeled dataset of interest. Common algorithms used for this purpose include k-nearest neighbors [64], Support Vector Machine (SVM) [65], and various deep learning models [66,67]. Alternatively, researchers can also use other well-annotated SG scRNA-seq datasets to guide the annotation process.

Over the last several years, increasing attention has been directed towards the annotation and characterization of rare cells that populate the SG, particularly under pathological conditions, as these cells have been shown to play pivotal roles in disease progression. This is especially relevant for immune cell subtyping, as it can reveal insights into the delicate balance between pro- and anti-inflammatory states within the tissue microenvironment. Notably, there are a myriad of computational tools available today to facilitate cell identification and gene signature analysis, including SingleR [40], scType [68], the ModuleScore function from Seurat, and scCATCH [39]. It is worth noting that while considerable advancements have been made to these programs, limitations persist such as redundancy among gene sets which obscure small or weakly defined subpopulations or those that are marked by only a few marker genes [67]. To increase confidence in assigned cell identities, users should perform internal validation steps which may include evaluating score distributions across clusters and visualizing the expression of well-established marker genes within the annotated cell clusters [67].

Once cells are accurately annotated, the dataset can serve as a foundation for numerous downstream analyses, including differential gene expression, cell–cell communication mapping, and reconstruction of developmental or pathological trajectories. Several SG scRNA-seq studies have applied these computational tools to discover interesting genomic characteristics of the salivary glands. Nonetheless, critical questions remain regarding whether each dataset captures a sufficient number of cells, represents the full diversity of cell types, maintains adequate data quality, and can be reliably used for broader biological extrapolation. These challenges, along with key findings from existing studies, are discussed in the following section.

## 3. Applications of scRNA-Seq in Salivary Gland Research

### 3.1. Revealing Cellular Heterogeneity in the Adult Salivary Gland

Maintaining homeostasis is critical for the proper function of adult SGs, and scRNA-seq studies have provided a molecular framework to understand how mature cell types are maintained. The glandular epithelium consists of four principal cell types—acinar, ductal, myoepithelial, and basal cells. Acinar cells are responsible for producing and secreting saliva, while myoepithelial cells, through their contractile activity, move the saliva into the ductal network, where it is subsequently modified into a hypotonic fluid. Basal cells provide structural support to the ducts and maintain the various epithelial cell lineages [49]. The complexity of the SG architecture is underscored by the diversity of its cellular subpopulations. Three main acinar cell subtypes—serous [69,70,71,72], mucous [73,74,75,76], and seromucous—produce distinct salivary components. Serous acinar cells secrete enzymes such as amylase 1 (Amy1), whereas mucous acinar cells generate mucin-rich saliva; seromucous acinar cells secrete a combination of both. Similarly, ductal cells [77,78,79,80] include several subtypes: intercalated [81,82,83,84,85], granular convoluted tubule (GCT—rodent specific [86,87,88,89,90]), striated [91,92,93,94,95], and excretory [96,97]. Intercalated ducts participate in ion exchange and are thought to harbor stem cells, while GCTs synthesize and secrete growth factors such as Neural growth factor (Ngf) and Epidermal growth factor (Egf) [98,99]. Striated ducts are central to ion exchange and excretory ducts finalize and deliver hypotonic saliva to the oral cavity.

**Figure 2:**
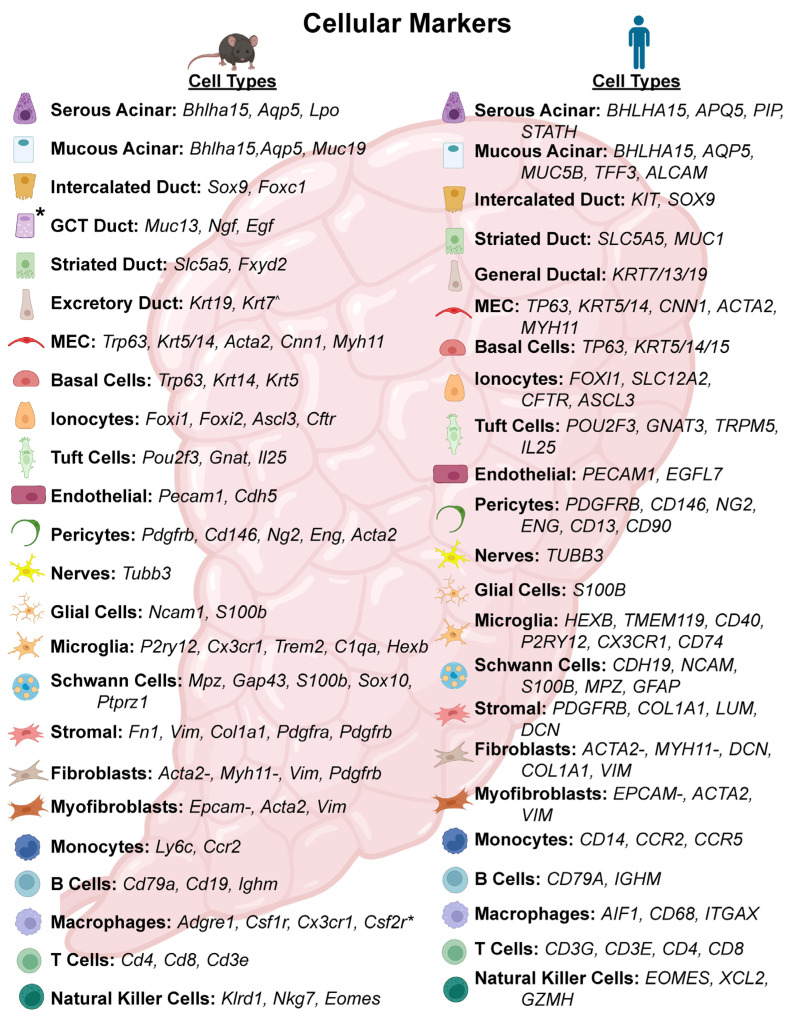
Established cellular markers for murine and human major salivary glands. Cell types within the major salivary glands are separated out by species with their respective known cellular markers. * = GCT (granular convoluted tubule) ducts are specific to rodents. ^ = General murine ductal marker. Images created in BioRender.

As alluded to in the previous section, earlier SG scRNA-seq studies were limited to identifying the more prevalent and previously characterized cell populations, but recent advancements in sequencing technology and improved dissociation protocols now enable the reliable detection of minor cell populations. Nonetheless, challenges remain as certain cell markers used in the murine SG are not interchangeable when evaluating the SG in the human context. For instance, Cystic fibrosis transmembrane conductance regulator (Cftr) marks exclusively the ductal cells in mice but is expressed in both acinar and ductal cell populations in humans [100]. To facilitate the annotation of human and murine scRNA-seq datasets, a curated list of established cellular markers enriched in the adult SG stratified by species is provided in Figure 2 and Supplementary Table S2. However, it is worth noting that certain rare cell populations remain incompletely characterized, and their identifiable cellular markers have yet to be fully defined. Additional variation also arises from differences among the major SGs [4] and between sexes in mice [32,86]. For example, *Amy1* is expressed in the acinar cells of the SMG and PG but is largely absent in the SLG. Similarly, sexually dimorphic genes have been identified in young male and female murine SMGs, with a notable increase of *Smgc* in female glands [69]. Bulk RNA-seq studies have already begun to reveal transcriptional diversity across glands which can be further pursued at a more granular level by scRNA-seq studies [101].

With the expansion of information concerning cellular markers, scRNA-seq has revealed the intricate landscape of both human and murine SGs. For instance, datasets of adult male and female human SMGs have delineated the diverse epithelial and non-epithelial cell populations, including human-specific mucous acinar cells and rare ionocytes [70]. A parallel study of adult human PGs explored the cellular heterogeneity and molecular mechanisms at a single-cell resolution [71]. Given that the PG is the largest SG in humans and produces the majority of stimulated saliva, these findings are particularly significant [3]. Chen et al. further examined PG acinar cells and identified enriched genes such as *AMY2A* and salivary peptides including statherin and histatins [71]. Interestingly, basal cells were not detected in the human PG dataset, although they are readily observed in the murine PG, underscoring the need for deeper examination of human samples to detect the wide range of sub-ductal populations and potentially other missing cell types [102]. Comparative studies between human and murine PGs at single-cell resolution would also help delineate conserved and species-specific differences.

One key limitation in the SG field is the lack of definitive cellular markers for distinct epithelial cell populations. Chen et al. leveraged their dataset of a healthy human PG together with a minor salivary gland scRNA-seq dataset [103] to compare the transcriptomes of serous acinar cells of the PG to mucous acinar cells of the minor salivary glands in order to distinguish markers between the subtypes of acini. Their analysis revealed parotid gland-specific serous acini markers—*HTN1* (Histatin 1) and *LTF* (Lactotransferrin). Additional studies in murine SGs identified angiotensinogen and galanin as novel markers of serous and mucous acinar cells, respectively [104], although experimental validation is necessary to confirm these findings. Indeed, follow-up confirmatory studies will be critical to improving our understanding of acinar cell subtypes. Along the same line, subtypes of other cell populations have been identified, as exemplified by two distinct murine intercalated ductal subpopulations marked by *Gstt1* (Glutathione S-transferase theta 1) and *Gfra3* (GDNF family receptor alpha 3) [69]. Recent studies from the Baker laboratory have revealed a rare population of tuft cells in the SMGs of various species (i.e., mouse, pig, and human) using transmission electron microscopy and confocal immunofluorescent analysis for markers [105]. These cells have been previously shown to promote inflammation in other tissues [106], and thus may be involved in inflammatory responses in the SG. Interestingly, tuft cells have also been implicated in tissue regeneration, as they can survive irradiation and contribute to epithelial renewal in the intestines [107]. Despite growing evidence for the presence of tuft cells in the SG, scRNA-seq-based detection of such cell types has been challenging, likely due to their rarity.

Although much attention has centered on epithelial cells, non-epithelial components, including nerves [108,109,110,111,112,113,114,115], vasculature [116,117,118,119,120], and immune cells [121,122,123,124,125,126,127,128,129,130,131,132,133,134,135,136,137], are equally vital in the normal and diseased state of the SG. Parasympathetic and sympathetic innervation governs saliva flow, with muscarinic receptor activation promoting saliva secretion and adrenergic signaling inducing protein-rich saliva secretion [138,139]. Neural networks interface with both epithelial and endothelial cells, with the latter ensuring nutrient delivery to the SGs. However, neuronal populations of the SG remain relatively unexplored at the single-cell level, likely due to the fact that most dissociation protocols focus on epithelial cell enrichment. A recent transcriptomic study that prioritized other cell populations pooled murine SMG and SLG tissues and found that endothelial cells in the stroma corresponded to capillary subtypes [116]. Comparative analyses across glands may reveal how endothelial subtypes and their secreted factors differ regionally [140]. Interestingly, SG endothelial cells express unique gene signatures [116] not shared by other tissues, suggesting specialized roles within the SG microenvironment. Additionally, immune cells such as B cells, T cells, dendritic cells, and macrophages play a role in immune surveillance under homeostatic conditions and during pathogenic states [141,142]. However, some immune cell populations like neutrophils are challenging to capture for scRNA-seq due to their fragility during tissue dissociation.

Understanding immune cell dynamics under homeostatic and diseased states also remains a priority. Under homeostatic conditions, macrophages interact with epithelial progenitors and endothelial cells; however, after radiation injury a subset of Csf2r^+^ (colony-stimulating factor 2 receptor) resident macrophages, which are capable of supporting salivary gland function, are depleted [126]. These Csf2r^+^ murine macrophages secrete Hgf (Hepatocyte growth factor), which has been shown to regulate epithelial progenitor cells and promote vascularization. Such interactions could facilitate the glandular regeneration program if these macrophages can be specifically targeted. However, despite these promising findings, it remains essential to validate whether an equivalent macrophage subset exists in adult human SGs.

While much of the foundational work in the SG has relied on murine models, the need to better understand human-specific biology has driven the development of advanced experimental systems. Among these, organoids have emerged as a transformative model, enabling the reconstruction of three-dimensional structures that closely mimic the architecture and function of native human glands. These models provide a powerful platform for investigating disease mechanisms, regenerative processes, and therapeutic responses in a physiologically relevant context. Initial studies of organoids were limited by inconsistent expression of lineage markers and incomplete recapitulation of in vivo organization. However, significant progress has been made through the optimization of extracellular matrix (ECM) composition and scaffold design [74]. Notably the transition from Matrigel-based to collagen-based has addressed issues related to immunogenicity and undefined matrix composition, moving the field closer to clinically applicable models. In an important step forward, Jeon et al. performed scRNA-seq analysis on human SMG tissue and human salivary gland organoids (hSGOs) cultured in Matrigel-based conditions that prolonged the proliferative phase [143]. Their studies revealed an expansion of actively proliferating cells and a predominance of ductal-like cells which are known to harbor stem cells in their native in vivo environment. Further optimization using collagen-based matrices enabled successful engraftment of hSGOs into irradiated mice, with increased mucin production providing functional evidence supporting organoid-based therapeutic strategies for xerostomia. Further evidence was also observed when chemically defined, Matrigel free hSGOs were engrafted into a Sjogren’s disease (SjD) mouse model restoring some stimulated saliva producing capabilities [55]. Importantly, these organoids demonstrated that under Matrigel-free conditions, long-term growth of mature acinar cells was supported and illustrated the composition of multiple epithelial cell lineages through scRNA-seq [55]. Lineage tracing using the hSGO scRNA-seq data revealed that basal type cells diverged toward ductal- and acinar-like cell types [55] which corresponds well to native salivary gland data indicating p63^+^ basal cells can differentiate into these cell populations [8]. Organoids have also proven to be a useful and pliable model system to evaluate molecular mechanisms and changes in cell populations upon irradiation treatment as reported recently [144]. In this elegant study, Cinat et al. used scRNA-seq to investigate the cellular and molecular responses of SG organoids to photon and proton irradiation and in the process unearthed a pro-regenerative role of IFN-I (type I interferon) signaling in the SG [144].

While these developments represent major progress, it is important to further our understanding of the fundamental similarities and distinctions between human SG organoids and native human SG tissue. Encouragingly, scRNA-seq studies have been recently conducted on organoids presenting all three major SGs [74]. A comparative examination of SG organoids and tissue revealed a high degree of transcriptomic similarities; however, *Amy1*, a highly expressed gene in SG scRNA-seq datasets, was barely detectable in the organoids, highlighting a shortcoming of this in vitro model system [74]. Further extrapolation of these datasets could be useful for a better understanding of SG biology, particularly mechanisms for the stem and progenitor cell functions.

### 3.2. Transcriptomics of the Developing Salivary Gland

Human and rodent SGs follow parallel developmental trajectories and share many similar biological processes, making rodent models, particularly mice, valuable systems for translational research. SG morphogenesis proceeds through a series of distinct developmental stages that are largely conserved across species. The process begins with epithelial thickening into a placode, which appears around embryonic day 11.5 (E11.5) in mice and during the 5th intrauterine week in humans [4,145]. Guided by epithelial–mesenchymal interactions, the placode invaginates into the underlying mesenchyme to form a primary stalk and initial bud [146,147,148,149]. This nascent structure undergoes repeated rounds of clefting, a process known as branching morphogenesis [148]. During the canalicular stage, ductal lumenization begins and the main epithelial cell types emerge, although recent evidence suggests that lineage cell fate decisions may be initiated even earlier [150]. The final, or terminal bud stage, marks near-complete lumenization of ducts and acinar end buds permitting continuous salivary flow. Postnatal maturation continues until puberty, at which point the granulated convoluted tubules form in mice [86].

Single-cell transcriptomic studies have been performed during SG development in both human [151] and mouse [69], offering an unprecedented close up view of transcriptomic changes during cellular differentiation and lineage commitment. These insights are particularly valuable for regenerative medicine where understanding the molecular cues that drive acinar cell differentiation is of central importance, as acinar cells are among the most vulnerable in diseased states [152,153,154,155,156]. Among various approaches, scRNA-seq has emerged as a transformative tool, enabling the identification of key regulators of differentiation in both human and mice [69,151,157].

Acinar cells originate in the end buds during development, where distinct cellular clusters can be readily detected by E14 in mice. Interestingly, the end bud cell population can be sub-clustered based on transcriptional differences between the inner and outer layer. Indeed, a sub-population of end bud cells demarcated by Actg1 (Actin gamma 1) or Cldn10 (Claudin 10) in an E14 SMG scRNA-seq dataset was shown to mark the inner or outer layer of the end bud, respectively [69]. These early transcriptional differences between the inner and outer end bud layer likely reflect lineage specification towards pro-acinar cell fates. Using the murine single-cell developmental atlas [69], Chatzeli and colleagues further characterized the inner and outer end bud layers. The outer Krt14^+^ population exhibited high Notch signaling activity, whereas the inner layer of Krt18^+^ cells showed low Notch signaling, highlighting how spatially restricted signaling events may shape acinar lineage commitment [150].

Identifying transcription factors (TFs) responsible for the transition from pro-acinar cells to mature acinar cells remains a major focus. Early developmental datasets have detected pro-acinar cells as early as E16 in mice, while in humans, fetal scRNA-seq profiles show differentiation events in the distal tip (where pro-acinar cells are located) between 17 and 19 weeks of gestation [69,151,157]. Notably, BHLHE40 was enriched in the distal tip [151] and is known as a super-enhancer-associated gene expressed at high levels in adult SGs compared to other tissues [158]. Additionally, while several TFs have been shown to contribute to the acinar cell lineage such as Sox2 [159], correlation analyses of postnatal day (P1) scRNA-seq SMG datasets revealed multiple genes associated with Bhlha15 [160,161,162,163,164] (a mature acinar cell marker) including Creb3l1, Spdef, Etv1, Creb3l4, Ehf, and Xbp1 [69]. These findings parallel other organ systems where Spdef promotes goblet and Paneth cell differentiation in lung and gut, respectively—cell types with secretory functions analogous to SG acinar cells [165,166], and Xbp1 which acts as a conserved regulator of secretory cell identity [167]. While these candidates provide promising leads, the precise mechanisms by which they orchestrate acinar maturation and whether they differ between mucous and serous subtypes remain unresolved.

Beyond cell intrinsic mechanisms, epithelial–mesenchymal signaling and crosstalk play a pivotal role in shaping both morphology and cell fate. Tissue recombination experiments have highlighted the importance of mesenchymal support [168,169,170], but more recent studies have revealed the PDGFRα^+^ stromal population as a critical niche component [157,171,172]. scRNA-seq analyses have shown that PDGFRα^+^ stromal cells exposed to FGF2 upregulated BMP7 which subsequently induced Aqp5 expression, a pro-acinar cell marker [157]. This subset of stromal cells expresses multiple components of the FGF pathway, suggesting various mechanisms at play including those promoting epithelial cell maturation [157,173]. Moreover, trajectory analyses, which computationally infer the developmental progression and lineage relationships among single cells based on transcriptional similarity, indicated that PDGFRα^+^ stromal cells possess the potential to give rise not only to pro-acinar cells but also to other lineages, emphasizing their multipotent influence on gland development [157].

While acinar cell differentiation has received much attention over the years, scRNA-seq studies have begun to reveal molecular subtypes of other epithelial cell lineages that were previously unappreciated. Furthermore, previously unrecognized sex differences have emerged—Serpin Family B Member 11 (Serpinb11) enrichment in male and Doublecortin Domain Containing 2a (Dcdc2a) in female murine intercalated duct—that can be detected as early as P20 and become more evident as the gland continues to grow and sexual dimorphism becomes more apparent [69]. Similarly, fetal datasets identified a subpopulation of MECs marked by LGR6, hinting at the possibility that additional, functionally distinct cell types may exist within the developing gland [151]. Limited recovery of MECs during isolation, however, remains a technical barrier to further granular resolution of this cell population.

Successful SG morphogenesis depends on support from surrounding cell types as ablation of vascular [117] or neuronal inputs [174] disrupts branching morphogenesis and alters epithelial patterning, highlighting the importance of these networks. Moreover, inhibition of vascularization, for instance, reduces Kit^+^ progenitor cells and increases ductal marker expression within the end buds [117], while parasympathetic denervation results in diminished K5^+^ progenitor cell populations and reduced branching [174]. Despite their importance, non-epithelial supporting cell populations including nerves and mesenchymal cells remain relatively underexplored by single-cell analyses, leaving much to be learned about intercellular signaling in gland development. In order to help rectify this situation, cell–cell communication analysis was performed on embryonic SMG evaluating the signaling patterns from a variety of supporting cell populations including neuronal, immune, endothelial, and mesenchymal to epithelial cells at E16 [175]. To date, most developmental single-cell studies have focused on the SMG, with fewer datasets available for the SLG and PG (Supplementary Table S1). However, one comparative study examining E12 mesenchymal cells from the PG and SMG revealed distinct expression signatures related to neuronal and muscle cell lineages, suggesting early spatial patterning differences that may influence adult gland structure and function [176].

Traditional in vivo and in vitro models have revealed critical mechanisms governing epithelial–mesenchymal interactions, signaling dynamics, and lineage specification. However, the scope of these studies remains limited compared to single-cell approaches, which can capture cellular heterogeneity, pathway activation, and lineage trajectories within a single experiment. A prime example is a scRNA-seq-based study evaluating the fluctuation of different signaling pathways amongst the diverse cell populations during development which ultimately exposed an understudied signaling pathway known as Midkine shown to contribute to the branched structure of the gland [175]. Nonetheless, many scRNA-seq studies underutilize available analytical tools such as trajectory inference or cross-species comparison. Expanding on such analyses will be essential to bridge murine and human developmental paradigms. Importantly, recent fetal human SG single-cell datasets have validated several mechanisms identified in mice—such as FGF and WNT spatial patterning—while revealing human-specific features including chEA3 expression in ductal cells and a novel stem-like population within striated ducts with acinar plasticity [151].

In sum, the integration of classical developmental biology and genetics-based approaches with emerging single-cell technologies is transforming our understanding of SG morphogenesis. Although considerable progress has been made in delineating the molecular mechanisms driving cell differentiation, many fundamental questions, particularly those concerning lineage plasticity, cross-species conservation, and the full extent of epithelial–mesenchymal–neural crosstalk, remain open for exploration.

### 3.3. Age-Related Salivary Gland Dysfunction

The tissue environment and cellular ecosystem of the SGs undergo profound changes with age, leading to progressive functional decline. Hallmarks of this process include increased inflammatory activity, fibrosis and adipose tissue accumulation, architectural disorganization, cellular senescence, and reduced saliva production [177,178]. Despite these well-recognized physiological changes, the molecular interactions among aging cell populations remain insufficiently characterized. A recent comprehensive scRNA-seq study has shed light on the transcriptional landscape of adult versus aged murine SMGs [179]. The findings from this study confirmed previously reported trends, such as loss of the acinar cells with age, but also revealed other nuanced yet important changes. Notably, basal and myoepithelial cell populations, key progenitor cell populations vital for maintaining salivary gland homeostasis, were also diminished in aged tissues [179]. Moreover, genes involved in major histocompatibility complex (MHC) class I antigen processing were upregulated in ductal cells, suggesting an age-associated activation of immune pathways that may represent novel therapeutic targets [179]. Interestingly, similar MHC class I related immune signatures have been observed in ductal cells from patients with Sjögren’s Disease (SjD), suggesting a potential mechanistic overlap between aging and autoimmune conditions [180]. While this study provided important insights to the field, not all acinar or ductal subtypes could be resolved, underscoring the need for deeper and more refined single-cell profiling of aging SGs.

### 3.4. Unraveling the Complexity of SG Cancer and Other Diseased States

SG dysfunction can arise from diverse insults including cancer, radiation exposure, and autoimmune diseases. Among these conditions, pleomorphic adenoma (PA) represents the most prevalent SG tumor occurring mainly in the PG and is characterized by pronounced histological and molecular heterogeneity [181,182,183,184]. Although scRNA-seq studies in the context of SG cancer remain limited, those focused on PA have begun to unravel the underlying molecular and cellular complexities at unprecedented resolution. In one study, tumor-initiating CD36^+^ myoepithelial cells were identified in PA patients as potential therapeutic targets, with inhibition of the PI3K-AKT signaling pathway suppressing their oncogenic activity [185]. A complementary study implicated cytoskeleton-remodeling genes and the transcription factor, FOXC1, as a potentially important regulator of PA progression [186]. These findings dovetail well with the key role of FOXC1 as a driver for organ-inductive signals [83] and the fact that FOXC1 is associated with crucial gene regulatory super-enhancer regions in the murine [158] and human [187] salivary tissue. Future validation using organoid models and loss-of-function approaches will be essential to clarify these mechanisms.

Salivary adenoid cystic carcinoma (ACC), though less common than PA, accounts for 10% of all SG malignancies and poses significant clinical challenges and high recurrence rates [188]. ACC is characterized by elevated MYB expression, which promotes proliferation and activates Notch signaling [189]. A recent study encompassing scRNA-seq analyses revealed that malignant transformation involves both luminal cells and MEC cell lineages with ACC tumor progression driven by cross-talk between Notch ligands in MECs and Notch receptors in luminal cells [190]. Expanding on these findings through cell–cell communication analyses could further elucidate the signaling networks that sustain ACC pathology.

Therapeutic options for advanced SG cancers remain limited. Surgical intervention is not always feasible, and immune checkpoint inhibitors show inconsistent efficacy [191,192,193]. To better define the immune landscape, single-nuclei RNA-seq profiling of 13 SG patient samples was undertaken in a recent study [192]. These studies, which included inflammation-high and inflammation-low ACC and non-ACC tumors [192], led to a detailed characterization of tumor-associated immune populations and revealed a predominance of M2 polarized macrophages, followed by T-cells. Since M2 macrophages promote immune checkpoint inhibitors (ICI) resistance, identifying other therapeutic targets is critical. A particularly promising candidate ripe for follow-up is VCTN1, found to be upregulated in ACC tumor cells and potentially linked to poor ICI responsiveness [192]. Taken together, these studies have set the stage for future investigations which should incorporate broader patient metadata, including irradiation history, viral exposure, such as SV40 [181], age, and sex, as these variables may influence tumor behavior and treatment outcome.

### 3.5. SG Injury Caused by Radiation Therapy

Radiation therapy, commonly administered for head and neck cancer, frequently leads to irreversible SG damage and xerostomia [194]. Both human and animal studies have documented marked acinar cell loss and reduced saliva production following irradiation [155,194]. Importantly, scRNA-seq analyses [195] have revealed the nature of injury with chronic exposure models where low-dosage exposure allows partial acinar survival but still compromises gland function. Rheinheimer and colleagues conducted an in-depth scRNA-seq analysis of chronically irradiated murine PGs, uncovering stress-associated transcriptional programs in acinar cells involving neurotrophin, neuregulin, and immune signaling pathways [195]. The identification of alterations to neuregulin is particularly interesting given its well-established role in SG development [196]. Notably, while dysregulation of neurotrophin signaling in MECs has been linked to SG dysfunction [109], other models have shown it may have a protective effect on acinar cells [195]. These findings emphasize the intricate and sometimes opposing functions of neurotropic signaling under injury conditions. A previously unrecognized population of secretory and ductal cells expressing Etv1 (ETS variant transcription factor 1) was also identified, representing a potential transitional state that may contribute to regeneration [195]. Etv1^+^ cells were enriched for neuregulin pathway activity, supporting a potential regenerative role that warrants further experimental validation [197,198]. However, this study also highlighted technical challenges in SG dissociation, as critical progenitor basal cell types capable of replenishing multiple epithelial cell lineages [8]—were not recovered in the scRNA-seq analyses. Future work should address this gap to better understand how radiation affects basal cell dynamics and regenerative potential.

Beyond the epithelial compartment, non-epithelial support cells in the SG also exhibit marked alterations after irradiation. While macrophage and endothelial numbers decline, T cell infiltration increases upon radiation damage, suggesting an unchecked immune response that may exacerbate post-radiation dysfunction [195]. Under homeostatic conditions, macrophages and endothelial cells are major sources for Sphingosine-1-phosphate, a sphingolipid, which provides protection for irradiated-treated murine SGs if pretreatment is administered [199]. A more complete mechanistic understanding of these immune/endothelial–epithelial interactions will be essential for designing strategies to restore glandular integrity and function following radiation injury.

Although radiation injury can reduce macrophage abundance. Hedgehog signaling has been shown to restore this population post radiation [200]. Delivery of Sonic hedgehog (Shh) via adenoviral vectors to murine SMGs upregulated Hedgehog pathways signaling components in progenitor cell populations and endothelial cells, and increased expression of macrophage-derived factors such as C1q, Csf1, and IL34 signaling across several cell populations [200]. Collectively these findings indicate that transient Hedgehog activation can stimulate the Csf1/IL34-Csf1r signaling axis to facilitate SG recovery post-irradiation [200]. Interestingly, shifts in macrophage subsets have also been observed in an acute radiation model. In these studies, proliferating Csf1r^+^ macrophages declined at the height of injury, and macrophage depletion prior to radiation resulted in pronounced structural abnormalities within the SG [128]. Together, these data underscore the indispensable role of macrophages in maintaining and restoring glandular integrity. Future studies should build upon this work to clarify additional macrophage-derived signals that support SG regeneration, particularly in damaged tissues.

### 3.6. Transcriptomic Deviations Induced by Viral Insults in the SG

Viral infections represent an important yet underexplored cause of SG dysfunction. Recent attention has focused on how SARS-CoV-2 (COVID-19) affects SG biology, as the oral cavity serves as a key site of viral entry and transmission [103]. Clinical manifestations of COVID-19 infection, such as xerostomia, altered taste perception, and oral mucosal lesions, suggest SG involvement may contribute to these symptoms; however, long-term consequences remain unclear [201]. scRNA-seq analysis of human minor SGs has detected viral entry factors including ACE2 and TMPRSS, confirming that SGs harbor the molecular machinery required for SARS-CoV-2 infection, although only a limited number of ductal cells were captured in these datasets which might hint at the possibility of viral-induced damage to the ductal cell population in infected SGs [103]. Additionally, scRNA-seq of human organoids transfected with SARS-CoV-2 also showed expression of the viral entry factors and will serve as a useful model for future mechanistic studies [202]. Further interrogation of immune and epithelial cell transcriptomes in tissues of both acute and recovered patients could provide valuable insight into how COVID-19 potentially reshapes SG tissue homeostasis, repair, and overall function.

### 3.7. Obesity-Associated Alterations in Salivary Gland Biology

Increasing evidence indicates that obesity also impairs SG structure and function, leading to reduced gland weight, increased oxidative stress, inflammatory remodeling, and diminished saliva secretion [203,204]. The transcriptional changes underpinning this condition are relatively ill-understood. A recent landmark study addressed this gap by performing scRNA-seq on SGs from high-fat diet-induced obese mice, including the first single-cell map of the sublingual gland [93]. Subsequent analyses revealed an expansion of immune cell populations, particularly T and B cells, across all major SGs. Using CellChat [205] the authors inferred differential intercellular communication networks between the glands, suggesting that obesity may uniquely influence each gland type [93], potentially due to inherent differences in their cellular composition [206]. For example, Annexin, App, and Thbs signaling were uniquely enriched in obese mice in the SMG, SLG, and PG, respectively, compared to the control glands [93]. In addition to mapping transcriptional alterations, the study proposed new molecular markers for sublingual gland cell populations [93], though confirmation of their expression at the protein level in vivo will be an important next step. Finally, single-cell analysis of human SGs in the context of obesity will be a future research area of high significance.

### 3.8. Advancing a Genetic Understanding of Autoimmune Diseases Impacting SG Function

Among autoimmune conditions involving the SG, such as IgG4-related disease [207] and SG sarcoidosis [208], Sjögren’s Disease (SjD) is one of the most common and debilitating. This chronic inflammatory disorder primarily targets exocrine glands such as the salivary and lacrimal glands, leading to loss of glandular function and severe dryness caused by abnormal immune activation and lymphocytic infiltration [209,210]. While the precise etiology of SjD remains unclear, several scRNA-seq studies have begun to shed light on the cellular and molecular mechanisms driving disease onset and progression. A consistently reported feature across human minor labial gland scRNA-seq datasets is the activation of the interferon (IFN) pathway, supporting earlier findings that were obtained from bulk RNA-seq and other experiments [131,132,211,212,213]. Interestingly, closer examination of these datasets indicates that IFN signaling is not restricted to immune cells but is also active in epithelial cell populations [212]. This supports the concept that epithelial cells may acquire a hybrid, “immune-like” phenotype that perpetuates chronic inflammation [209,214,215]. Comparable findings have been described in a well-characterized mouse model of SjD, where scRNA-seq analyses of control and diseased SMGs revealed a marked increase in immune-related genes, including IFN signatures, within both acinar and ductal cell compartments [180].

While these mouse models are invaluable when human samples are unavailable, it is important to recognize that they may not fully capture the heterogeneity of the human disease. Human SjD is a multifactorial heterogeneous disease shaped by genetic, pathogenic, and environmental influences, resulting in substantial patient-to-patient variability [216]. Nevertheless, a notable scRNA-seq study linked transcriptional profiles to specific autoantibody patterns (anti-SSA vs. anti-centromere) [131]. For example, TGFβ signaling was more prominent in anti-centromere-positive patients, whereas IFN signaling predominated in those patients positive for anti-SSA antibodies [131]. Differences were also observed in B cell subset expansion and gene activation profiles under distinct autoantibody conditions [131]. These distinctions align with the findings of Pranzatelli and colleagues, who reported enriched MHC-I signaling in SSA^+^ SjD salivary glands compared to SSA^−^ patient samples, as predicated by CellChat analysis [132]. Despite these variations, several core features remain consistent across studies of SjD. For instance, CCR1^hi^/CCL5^hi^ macrophages and CCL5^hi^ T cells have been identified in multiple SjD patient scRNA-seq datasets, and their predicted cell–cell communication networks suggest a contributory role in disease pathogenesis [217]. However, it should be noted that these conclusions were drawn from a limited number of patient datasets. In a more comprehensive analysis, Inamo et al. identified expanded GZMB^+^GNLY^+^ CD8^+^ T cells, followed by GZMK^+^ CD8^+^ T cells in human SjD SG across all scRNA-seq profiles [131]. Emerging evidence suggests that these GZMK^+^ CD8^+^ T cells may target a subset of acinar cells [132], potentially explaining the consistent loss of seromucous acinar cells observed in human SjD scRNA-seq datasets [132,212], an observation that is also mirrored in the murine model [180]. Furthermore, human SjD acinar cells exhibit elevated JAK and STAT pathway gene expression, indicative of an inflammatory state, which can be mitigated by JAK inhibition in cultured primary salivary gland epithelial cells [212].

Although much attention has focused on immune–epithelial interactions, other cell types also contribute to SjD pathogenesis. For example, endothelial cells demonstrate heightened IFN signaling [132] and an ACKR1^+^ endothelial cell subset has shown upregulation of chemokine-associated genes that may facilitate lymphocyte transendothelial migration (TEM) [211]. Fibroblasts represent another key cell population which have been reported to influence the formation of tertiary lymphoid structure (TLS) and overall disease progression [218]. CellChat-based analyses have revealed that cell–cell communication patterns are profoundly altered in the disease, with enhanced interactions with B cells, CD4^+^ and CD8^+^ T cells, and reduced interactions with epithelial cells [211]. Downregulated fibroblast-associated pathways were linked to extracellular matrix organization and WNT signaling, potentially impairing epithelial regeneration [211]. A THY1^+^ fibroblast subset, enriched for genes associated with lymphocytic recruitment, was found in close spatial proximity to immune cells, suggesting a role in amplifying local inflammation [131]. Finally, pericytes, though often underrepresented in scRNA-seq datasets, exhibit a striking gain in immunogenicity in SjD as these cells express chemokines such as CCL21 and CCL19, along with proinflammatory genes, underscoring their potential involvement in disease mechanisms [219]. Collectively, these integrated datasets establish a valuable framework for identifying therapeutic targets and deepening our molecular understanding of SjD.

### 3.9. Gland Regeneration

While disease models have been instrumental in elucidating the underlying causes of SG dysfunction, regenerative studies offer a complementary perspective by identifying the cellular programs and signaling pathways that promote tissue repair and restoration of function following injury. Organoids have proven to be one such powerful model system for such investigations, as they faithfully recapitulate the major SG cell populations [74,220,221] and can be experimentally manipulated to model various insults or to test candidate modulators of repair. The addition of scRNA-seq to these models provides an unprecedented layer of resolution. For instance, Cinat et al. applied scRNA-seq to irradiated and non-irradiated SG organoids and found that progenitor cell populations were enriched for genes involved in development and morphogenesis following irradiation, consistent with self-renewal capacity [222]. Notch signaling was notably upregulated in these progenitor cell subsets, and functional assays confirmed its importance in maintaining self-renewal capabilities [222].

Another widely used approach for studying tissue injury and regeneration is SG ductal ligation, a reversible injury in the murine model. An elegant study performed by the Larsen laboratory employed ductal ligation to characterize transcriptional changes in endothelial cells during injury and recovery using scRNA-seq [116]. Their results show that endothelial cells from the ligated gland exhibit a pro-fibrotic profile and signs of endothelial-to-mesenchymal transition (endoMT), whereas cells from regenerating glands express angiocrine factors thought to promote tissue repair. Ligand–receptor interaction analyses further revealed that the Ntf3-Ntrk2 pair, which is part of the neutrophin-signaling pathway, was among the most enriched during injury, suggesting a context-dependent role for neutrophin signaling in vascular remodeling and regeneration [116].

The ductal ligation model has further been used to study fibrosis—a common outcome of ductal obstruction, aging, disease, and radiation [194,223,224,225,226,227]. Persistent fibrosis leads to loss of function, cell death, and acinar cell atrophy. Altrieth et al. employed scRNA-seq to examine stromal cell populations in wild-type mice subjected to ductal ligation after two weeks, focusing on Gli1, a transcription factor implicated in fibrosis across multiple organs [224]. Interestingly, ablation of Gli1 resulted in only a modest reduction in fibrosis, evidenced by decreased extracellular matrix deposition and collagen remodeling [224]. However, the scRNA-seq data suggested that a Pdgfrα/Pdgfrβ positive stromal subset might be the primary contributor to fibrosis [224]. Given that PDGFRα^+^ cell populations also promote pro-acinar cell differentiation during development, their dual role in fibrosis and regeneration warrants further investigation [157]. Another scRNA-seq study conducted on pre-injury and 1, 3, and 7 days post-ductal ligation pooled SMG/SLG identified three major groups of fibroblasts [228]. Interestingly, the authors found that distinct groups of the fibroblasts showed dynamic gene expression profiles that were likely reflective of the distinctive functional properties during the tissue recovery after ligation-induced injury. These observations shed important light on transcriptional changes in the fibroblast populations during early stages of injury—such mechanistic insights can help develop better therapeutics and SG outcomes after injury.

Collectively, these studies underscore the importance of examining the complete cellular landscape when investigating SG injury and regeneration. Stromal cell [224,229,230,231,232] populations of the SG, including endothelial cells, macrophages, and fibroblasts, are emerging as critical players in the repair and regenerative programs. Moving forward, attention should also be given to the contribution of rare or underrepresented populations, such as pericytes and tuft cells, which are often missed due to limited sequencing depth or sampling size. A comprehensive understanding of these cell types and their interactions will be essential for developing targeted regenerative therapies for SG dysfunction.

## 4. Perspectives and Future Directions

### 4.1. Challenges

**Figure 3:**
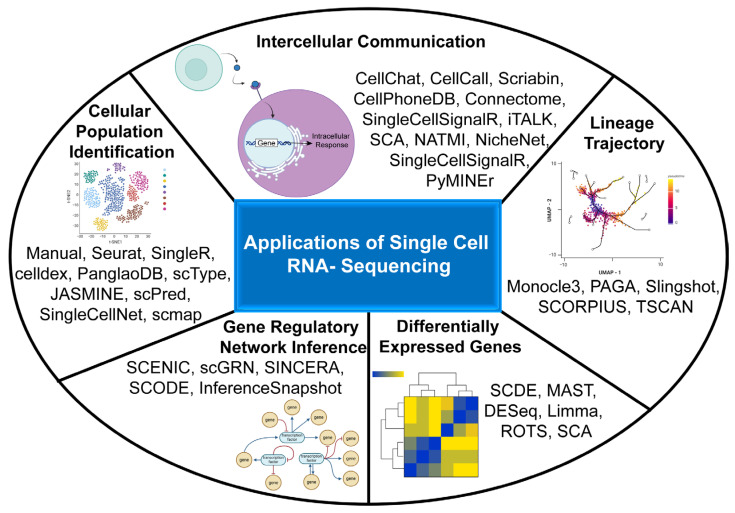
Sophisticated computational tools for downstream scRNA-seq analysis. Graphic illustration summarizing the various downstream computational applications available for scRNA-seq data with their respective programs. Images created in BioRender.

A central goal of SG scRNA-seq-based studies has been to map the diversity of resident cell populations and uncover transcriptomic differences between physiological and diseased states. While these efforts have greatly advanced our understanding of SG biology, key analytical opportunities remain underutilized (Figure 3). Notably, only a limited number of studies have explored cellular trajectories [69,151] or performed in-depth cell–cell communication (CCC) analyses [70,116]. Single-cell trajectory analysis provides a window into the dynamic continuum of cellular transitions, such as differentiation or injury responses, while CCC analysis predicts intercellular signaling events that shape developmental programs, maintain tissue homeostasis, and influence disease progression. Several robust computational tools such as CellChat [205], CellPhoneDB [233], and Connectome [234] are available to interrogate these interactions and identify candidate pathways for therapeutic intervention. The salivary epithelium, in particular, exists within a complex microenvironment that relies on interactions with mesenchymal and stromal cells during both organogenesis and repair [149,235]. Thus, applying CCC analyses to developmental or injury datasets could help delineate how specific signaling networks drive epithelial differentiation and regeneration.

However, many current datasets face practical constraints that limit such advanced analyses. In several studies, epithelial cells were underrepresented due to dissociation bias or sample composition. For instance, Chen et al. analyzed over 16,000 cells to generate the first transcriptional profile of a healthy adult human male PG [71]. However, the dataset was largely dominated by immune cells, and no ductal subtypes were identified. This limitation severely restricts the scope of downstream computational analyses to basic cluster identification and differential gene expression. On a similar theme, rare epithelial subtypes such as ionocytes [236,237,238,239,240,241] or tuft cells [105,242,243,244] are frequently absent or overlooked in scRNA-seq studies that are not sufficiently powered in cell numbers and sequencing depth. Ionocytes were first characterized in adult murine SGs [236] using scRNA-seq [69], and their presence was later confirmed in human SMGs by Horeth et al. [70]. These cells are of particular interest given their expression of FGF10, a factor critical for epithelial development and their potential use in regenerative strategies. Similarly, tuft cells, recognized by their distinctive apical microvilli and expression of the transcription factor POU2F3 [105,245,246,247,248], serve chemosensory and immunomodulatory roles [246]. Located within the striated ducts of both mouse and human SMGs [105,239,249], tuft cells may contribute to autoimmune pathologies such as SjD, where epithelial–immune crosstalk plays a pivotal role. Other rare, underrepresented populations include pericytes [250,251,252], which have been detected in murine stromal-enriched SG dataset [116] and a human SjD dataset [219] as well as goblet, Schwann [157,253,254,255], glial, and telocyte populations. As enzymatic dissociation, sequencing technologies, and computational tools improve, capturing these minor yet functionally important populations should become increasingly feasible.

Updating datasets with the latest versions of analytic pipelines, such as the commonly used 10X Genomics CellRanger or similar software, may also enhance cell recovery and improve annotation. However, such updates are dependent on data accessibility, and not all scRNA-seq datasets are publicly available through repositories like GEO (Gene Expression Omnibus). Moreover, missing metadata such as donor age or sex hampers interpretability and reproducibility. This is particularly relevant given that many human SG samples originate from older individuals, whose glandular transcriptomes and functions differ significantly from younger cohorts [177,178,226]. Despite these limitations, the scRNA-seq studies conducted to date have provided critical insight into SG development, immune involvement, and disease-associated transcriptional remodeling. Each dataset contributes to a growing framework for understanding the cellular and molecular basis of SG function and dysfunction.

### 4.2. Future Prospects for the SG Field

Although scRNA-seq has transformed the study of the SG, it inherently lacks spatial context and may inadvertently introduce transcriptional artifacts during tissue dissociation. Spatial transcriptomics (ST) addresses these challenges by preserving tissue architecture while maintaining gene expression resolution. This approach is especially valuable in disease settings such as Sjögren’s Disease [131,219], where immune infiltration occurs in close proximity to ductal structures, and understanding the spatial dynamics of these interactions is essential for identifying drivers of disease pathogenesis. Such studies are underway and a recent report containing a spatial transcriptomic dataset from submandibular glands in the IL-14αTG SjD mouse model should encourage similar investigations [256]. One caveat to keep in mind is that ST technology in its current iteration suffers from low transcript capture efficiency and a lack of true single-cell resolution, making integrative analysis with scRNA-seq, which provides higher resolution, an optimal strategy moving forward. A major advancement in this direction has come from a recent tour de force study that has generated an integrated single-cell and spatial proteotranscriptomics atlas of the human adult oral cavity that includes salivary gland [232]. Beyond spatial mapping, multi-omic approaches represent the next frontier in SG research. Integrating transcriptomic, epigenomic, proteomic, and metabolomic datasets provides a more comprehensive understanding of cellular identity, function, and mechanisms linking genotype and phenotype. Similar strategies have been successfully applied in other tissues, such as the skin, where multi-omic profiling has revealed cellular heterogeneity, disease-specific signatures, and therapeutic targets [257,258,259,260].

These advances underscore the potential of applying multi-omic integration to SG biology, ultimately paving the way for precision medicine approaches tailored to individual patients. Unfortunately, while similar single-cell resolution datasets are lacking in the SG, it is still possible to bridge the gap. For example, epigenomic datasets of adult human and murine SMGs [158,187], when coupled with single-cell transcriptomics, can reveal how chromatin states regulate gene expression patterns within specific cell populations. Similarly, CITE-seq now enables simultaneous quantification of cell surface proteins and mRNA, bridging the gap between transcriptomic signals and protein function [261]. Similarly, single-cell metabolomics analyses will further refine our understanding of cellular phenotypes. Encouragingly, bulk level studies integrating transcriptomic and metabolic data have already linked radiation-induced mitochondrial dysfunction to SG damage, foreshadowing the power of these combined datasets [262]. As analytical and computational tools advance, single-cell multi-omics will undoubtedly yield unprecedented insights into SG biology and pathology. The integration of these next-generation datasets will also intersect with the expanding role of AI, which is poised to accelerate discovery in data-rich biomedical fields. AI-driven models can enhance pattern recognition, predict signaling networks, and propose novel therapeutic targets—ushering in a new era of precision salivary gland research.

## 5. Conclusions

Single-cell transcriptomics have redefined our understanding of SG biology and disease, revealing previously underappreciated cellular heterogeneity and potential mechanisms of development, regeneration, and dysfunction. Continued progress, however, will depend on transparent reporting, complete metadata, and rigorous data integration. As computational tools evolve and scRNA-seq data are merged with spatial and other omic modalities, our view of the SG will become increasingly refined. Ultimately, these efforts hold the promise of translating molecular insight into novel and durable therapeutic strategies for salivary gland disorders.

## Data Availability

The original contributions presented in this study are included in the article/Supplementary Material. Further inquiries can be directed to the corresponding authors.

## Supplementary Materials

The following supporting information can be downloaded at: https://www.mdpi.com/article/10.3390/biology15080641/s1, Supplementary Figure S1. Overview of literature selection. Supplementary Table S1: Compilation of scRNA-seq datasets. Table containing key information about all the scRNA-seq datasets discussed in this review. Supplementary Table S2: References for cellular markers as identified in the adult salivary glands, as shown in Figure 2.

## Abbreviations

The following abbreviations are used in this manuscript:

scRNA-seq	Single-cell RNA-sequencing
SG	Salivary Gland
SMG	Submandibular Gland
PG	Parotid Gland
SLG	Sublingual Gland
FACS	Fluorescence-activated cell sorting
UMIs	Unique molecular identifiers
NGS	Next Generation Sequencing
SMRT	Single-molecule, real-time
QC	Quality control
MECs	Myoepithelial cells
SVM	Support vector machine
GCT	Granular convoluted tubule
Ngf	Neural growth factor
Egf	Epidermal growth factor
Cftr	Cystic fibrosis transmembrane conductance regulator
Amy1	Amylase 1
HTN1	Histatin 1
LTF	Lactotransferrin
Gstt1	Glutathione S-transferase theta 1
Gfra3	GDNF family receptor alpha 3
Csf2r	Colony stimulating factor 2 receptor
Hgf	Hepatocyte growth factor
ECM	Extracellular matrix
hSGOs	human salivary gland organoids
Actg1	Actin gamma 1
Cldn10	Claudin 10
TFs	Transcription factors
PA	Pleomorphic adenoma
ACC	Adenoid cystic carcinoma
ICI	Immune checkpoint inhibitors
Shh	Sonic hedgehog
SjD	Sjögren’s Disease
IFN	Interferon
TEM	Transendothelial migration
endoMT	Endothelial-to-mesenchymal transition
CCC	Cell–cell communication
GEO	Gene Expression Omnibus
AI	Artificial intelligence
