# Supplementary material for: A Mouthful of Genomic Data: Single-Cell Insights into Salivary Gland Biology and Disease
Source: Biology (Basel). 2026 Apr 18;15(8):641. doi: 10.3390/biology15080641 (PMC13113837; doi:10.3390/biology15080641)
Supplement: Supplementary file 1 [file biology-15-00641-s001.zip › Supplementary Figure S1.pdf]

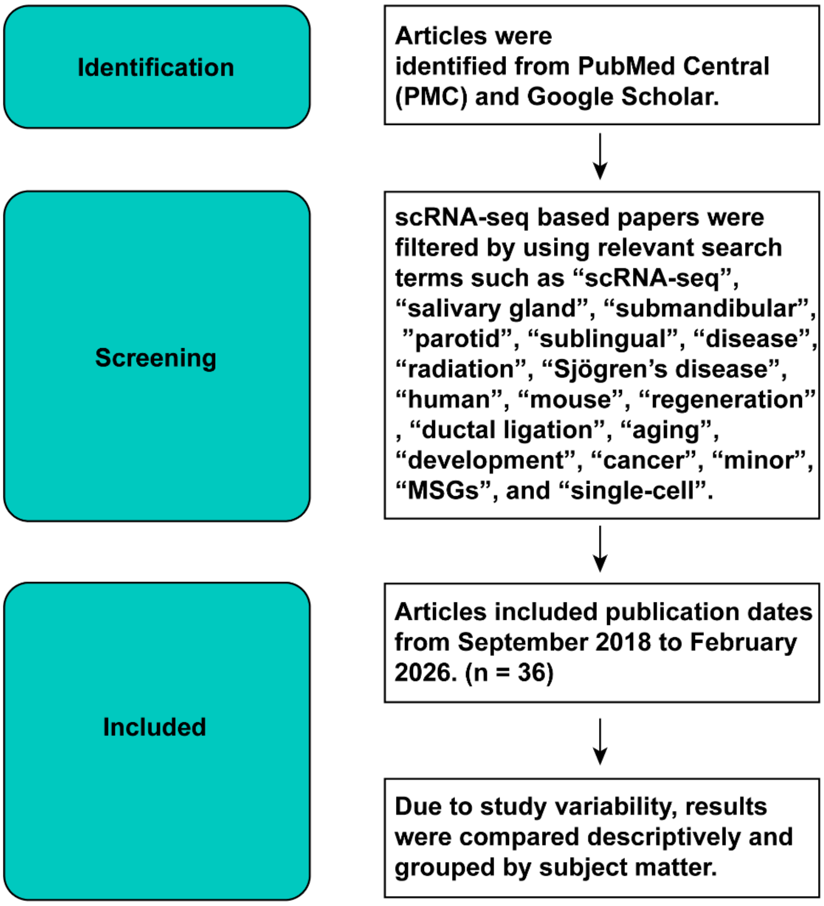

Supplementary Figure S1. Overview of literature selection
Workflow for identifying relevant scRNA-seq papers in the salivary gland field and description of how the review was organized.
