# Supplementary material for: A Mouthful of Genomic Data: Single-Cell Insights into Salivary Gland Biology and Disease
Source: Biology (Basel). 2026 Apr 18;15(8):641. doi: 10.3390/biology15080641 (PMC13113837; doi:10.3390/biology15080641)
Supplement: Supplementary file 1 [file biology-15-00641-s001.zip › Supplementary Table S2.pdf]

Supplementary Table S2. References for cellular markers as identified in the adult salivary glands as shown in Figure 2.

**Cell Type**	**Mouse**	**Human**
Serous/ Mucous Acinar	*Bhlha15* (Mist1)[160-162]	*BHLHA15* (MIST1)[163, 164]
Serous/ Mucous Acinar	*Aqp5*[8, 45, 46]	*AQP5*[47, 48]
Serous Acinar	*Lpo*[69]	*PIP*[70, 71]
Serous Acinar		*STATH*[70-72]
Mucous Acinar	*Muc19*[73, 74]	*MUC5B*[70, 72, 75]
Mucous Acinar		*TFF3*[70, 75]
Mucous Acinar		*ALCAM* (CD166)[70, 76]
Intercalated Duct	*Sox9*[81-84]	*SOX9*[70]
Intercalated Duct	*Foxc1*[83, 84]	*KIT*[70, 85]
GCT Duct	*Muc13*[84]	
GCT Duct	*Ngf*[87-89]	
GCT Duct	*Egf*[88-90]	
Striated Duct	*Slc5a5* (Nis)[69]	*SLC5A5* (NIS)[70, 91, 92]
Striated Duct	*Fxyd2*[93, 94]	*MUC1*[70, 95]
Excretory Duct	*Krt19*[96, 97]	
General Ductal	*Krt7*[77, 78]	*KRT7*[70, 79]
General Ductal		*KRT13*[96]
General Ductal		*KRT19*[70, 79, 80, 96]
MEC/Basal	*Trp63*[8, 49, 78]	*TP63*[50]
MEC/Basal	*Krt14*[51, 52, 78]	*KRT14*[50, 52, 53]
MEC/Basal	*Krt5*[51, 78]	*KRT5*[50, 53]
Basal		*KRT15*[55]
MEC	*Cnn1*[51]	*CNN1*[51]
MEC	*Acta2* (Sma)[51, 56]	*ACTA2* (SMA)[53]
MEC	*Myh11*[56]	*MYH11*[53, 57]
Ionocytes	*Foxi1*[236]	*FOXI1*[237-239]
Ionocytes	*Ascl3*[236, 240]	*ASCL3*[237, 238]
Ionocytes	*Cftr*[236, 240]	*CFTR*[237]
Ionocytes	*Foxi2*[236]	*SLC12A2*[70]
Tuft cells	*Pou2f3*[105, 242]	*POU2F3*[105, 239]
Tuft cells	*Gnat3*[242]	*GNAT3*[243]
Tuft cells	*Il25*[242]	*IL25*[243]
Tuft cells		*TRPM5*[243, 244]
Endothelial	*Pecam1*[116, 117]	*PECAM1*[118, 119]
Endothelial	*Cdh5*[116]	*EGFL7*[120]
Pericytes	*Pdgfrb*[250]	*PDGFRB*[250]
Pericytes	*Cd146*[250]	*CD146*[250]
Pericytes	*Ng2*[250]	*NG2*[251]
Pericytes	*Eng*[250]	*ENG*[250]

Pericytes	*Acta2* (Sma)[250]	*CD90*[251]
Pericytes		*CD13*[252]
Nerves	*Tubb3*[108]	*TUBB3*[109]
Glial cells	*S100b*[110, 111]	*S100B*[112]
Glial cells	*Ncam1*[69]	
Microglia	*P2ry12*[37]	*P2RY12*[113]
Microglia	*Cx3cr1*[37]	*CX3CR1*[113]
Microglia	*Hexb*[114]	*HEXB*[113]
Microglia	*C1qa*[115]	*TMEM119*[113]
Microglia	*Trem2*[115]	*CD40*[113]
Microglia		*CD74*[114]
Scwhann cells	*S100b*[253]	*S100B*[112]
Scwhann cells	*Mpz*[253]	*MPZ*[254]
Scwhann cells	*Gap43*[253]	*NCAM1*[255]
Scwhann cells	*Sox10*[253]	*CDH19*[254]
Scwhann cells	*Ptprz1*[157]	*GFAP*[254]
Stromal	*Fn1*[224]	
Stromal/Fibroblasts	*Vim*[224]	*LUM*[229]
Stromal/Fibroblasts	*Col1a1*[224]	*COL1A1*[230]
Stromal/Fibroblasts	*Pdgfra*[157, 224]	*DCN*[229]
Stromal/Fibroblasts	*Pdgrb*[157, 224]	*PDGFRB*[230]
Myofibroblasts	*Acta2*[224]	*ACTA2*[231, 232]
Myofibroblasts	*Vim*[224]	*VIM*[231]
Monocytes	*Ccr2*[121]	*CCR2*[122]
Monocytes	*Ly6c*[121]	*CCR5*[122]
Monocytes		*CD14*[122, 123]
B cells	*Cd79a*[37]	*CD79A*[70, 124]
B cells	*Ighm*[37]	*IGHM*[70]
B cells	*Cd19*[125]	*CD19*[70]
Macrophages	*Adgre1*[126, 127]	*AIF1*[37, 70]
Macrophages	*Csf1r*[128]	*CD68*[70]
Macrophages	*Cx3cr1*[128]	*ITGAX*[70]
Macrophages	*Csf2r*[126]	
T cells	*Cd4*[129]	*CD4*[130, 131]
T cells	*Cd8*[129]	*CD8*[130-132]
T cells	*Cd3e*[37, 133]	*CD3E*[70, 131]
T cells		*CD3G*[70]
Natural Killer	*Eomes*[134, 135]	*EOMES*[134]
Natural Killer	*Klrd1*[37]	*GZMH*[136]
Natural Killer	*Nkg7*[37]	*XCL2*[137]
